# Identifying, describing, and assessing interventions that support new graduate nurse transition into critical care nursing practice: a systematic review protocol

**DOI:** 10.1186/s13643-020-01483-7

**Published:** 2020-10-16

**Authors:** Brandi Vanderspank-Wright, Michelle Lalonde, Janet Squires, Ian D. Graham, Nikolaos Efstathiou, Robin Devey Burry, Emily Marcogliese, Becky Skidmore, Amanda Vandyk, Canadian Association of Critical Care Nurses and the National Emergency Nurses Association

**Affiliations:** 1 University of Ottawa, School of Nursing, 451 Smyth Road, Ottawa, Ontario K1H 8L1 Canada; 2 University of Ottawa, School of Epidemiology and Public Health, 600 Peter Morand Crescent, Ottawa, K1G 5Z3 Ontario Canada; 3 University of Birmingham, School of Nursing, Medical School, Vincent Drive, Birmingham, B15 2TT United Kingdom; 4 Bruyère Continuing Care, 60 Cambridge Street North, Ottawa, Ontario K1R 7A5 Canada; 5 Ottawa, Canada

**Keywords:** New graduate nurse, Transition, Intervention, New nurse, Critical care, Intensive care, Emergency department, Accident and emergency, Emergency room, Specialized unit

## Abstract

**Background:**

Given a persistent nursing shortage in Canada and a decline in new nurses entering the profession, new graduate nurses (NGNs) are being hired into positions historically reserved for more experienced staff. Critical care settings, which are areas of specialty nursing practice, are now routinely hiring NGNs in many hospitals. While evidence on NGN transition into critical care is emerging, best practices around training and support for these nurses are limited internationally, and non-existent within the Canadian context. Therefore, the *aim* of this systematic review is to identify, describe, and assess the effectiveness of interventions that support NGN transition into critical care clinical practice settings.

**Methods:**

This is a systematic review of interventions using the Joanna Briggs Institute Methodology. Data sources will include MEDLINE, CINAHL, PsychINFO, Education Source, and Nursing and Allied Health electronic databases. Two independent reviewers will screen titles and abstracts using predetermined inclusion criteria. A consensus meeting will be held with a third reviewer to resolve conflicts when necessary. Full texts will also be screened by two independent reviewers and with conflicts resolved by consensus. Data will be extracted using a standardized extraction form. We will assess the quality of all included studies using Joanna Briggs Institute quality assessment tools. Data describing interventions will be reported narratively and a meta-analysis will be conducted to determine effectiveness, if appropriate.

**Discussion:**

This systematic review will identify interventions that support NGN transition into critical care nursing practice. The findings of this study will provide a foundation for developing strategies to support NGN transition into these areas of specialty nursing practice.

**Systematic review registration:**

PROSPERO CRD42020147962.

## Background

In Canada, there is a shortage of practicing nurses [1] and limited human resources within the healthcare system. In 2017, the Canadian Institute for Health Information reported the first decline in new nurses entering the profession since 2008 [2]. In an effort to mitigate shortages and maintain sufficient human resources at the point of care, critical care settings, specifically intensive care units (ICU) and emergency departments (ED), are now routinely employing new graduate nurses (NGNs) in many hospitals [3, 4]. Today, nurses working in these critical care settings comprise the second largest body of nurses (15.7%) in a particular domain of practice [5].

NGNs hired into critical care settings are likely to encounter additional challenges as they *transition* into their nursing roles as they immediately require specialized training and knowledge to be able to practice safely [6]. In general, NGNs entering clinical practice experience a transition period of approximately 2 years. Over this period of time, they move iteratively through phases of doing, knowing, and being—in essence transitioning from a task-oriented practitioner to one with a solidified professional identity and able to provide whole person care. In many respects, the NGN transition into critical care is fundamentally different than transition into general nursing practice [7]. Critical care environments are fast paced and technologically advanced [8–10], and even *experienced* nurses working in these settings report high rates of moral distress because of the unpredictable nature of their work [11]. For NGNs, these challenges are compounded by complex ethical issues for which they are not prepared, such as medical futility, withdrawal or withholding of life-sustaining treatment, and medical aid in dying—though expected to manage [12, 13]. As a result, NGNs experience transition shock, which is described as feelings of disorientation, discouragement, and exhaustion. Transition shock can significantly impact successful transition and may result in burnout or even turnover (including intent to leave their position or the nursing profession entirely) [7, 14]. Turnover among NGNs is a notable problem; reports of 2-year turnover rates range from 35 to 62% [15–19]. More recently, Brewer, Kovner, Greene, Tukov-Shuser, and Djukic [20] reported a 1-year NGN turnover rate of 15.4% and a 3-year NGN turnover rate of 43.4%. Compared to the mean turnover rate of 20% for nurses generally [21], NGNs are nearly twice as likely to leave their current nursing position. In Canada and the USA, under preparation and lack of support for NGNs during their early work experiences are factors contributing to turnover [15, 22–24].

Two closely related synthesis studies were published within the past 5 years, demonstrating interest in supporting nurse transition into practice and the complexity of this process for new graduates seeking employment within critical care settings. First, Edwards and colleagues [22] conducted a systematic review to determine the effectiveness of the main strategies and interventions used to support newly graduated nurses’ transition into the clinical workplace generally [22]. The authors concluded that the use of transitional support strategies for NGNs demonstrate a beneficial effect with an overall positive impact irrespective of the type of support provided [22]. The authors included quantitative study designs only and restricted their search to English language papers [22]. The conclusion drawn that transitional support strategies generally for NGNs are beneficial positions our systematic review as relevant as we look specifically to the critical care setting. A more recent integrative study by Innes and Calleja [3] aimed to develop an understanding of the strategies that may inform the creation of an educational program to support the transition of nurses to critical care [3]. A constant comparison approach to thematic analysis of the data was used to develop six themes: having a designated resource person, workplace culture, socialization, knowledge and skill acquisition, orientation, and rotations [3]. Interestingly, the authors sought to understand existing transition support strategies as the basis of knowledge for the development of a critical care transition program, yet their inclusion criteria encompassed both acute and critical care. Therefore, conclusions drawn are not necessarily specific to the critical care setting despite this being implied in the title and body of the text. The article is limited, as acknowledged by the authors, by its exclusion of articles in languages other than English and its restriction to a 10-year timeframe. While evidence on NGN transition into critical care is emerging (e.g., [3, 25, 26]), best practices around training and support are limited internationally and non-existent within the Canadian context. Therefore, the aim of our systematic review is to identify, describe, and assess the effectiveness of interventions that support NGN transition into critical care clinical practice.

## Methods

### Design

This is a systematic review of effectiveness guided by the Joanna Briggs Institute Methodology for Reviews of Effectiveness [27].

### Protocol and registration

This protocol was prepared according to the Preferred Reporting Items for Systematic Reviews and Meta-Analyses Protocols (PRISMA-P checklist) [28]. The PRISMA-P checklist is included as an additional file (see Additional file 1). This protocol is registered with PROSPERO (CRD42020147962).

### Eligibility criteria

Eligibility criteria were created using PICO (**P**opulation, **I**ntervention, **C**omparison, **O**utcomes), as specified in the Joanna Briggs Institute methodology:

**P:** New graduate nurses; **I:** All forms of interventions about transitioning NGNs into critical care clinical practice. *Critical care* will be defined as any general medical and/or surgical intensive care unit (neonatal, pediatric, adult, or combination thereof) including specialized units (e.g., neurosurgical intensive care unit, cardiovascular intensive care unit), as well as any emergency department.; **C:** Other or no intervention; **O:** Operational definition of “new graduate nurse,” inventory of interventions (identify), summary of intervention characteristics (describe), and reported results (assess effectiveness). Reported results will include effect on NGN transition (statistical or narrative) and other author-identified outcomes.

We will include quantitative (experimental and non-experimental) and qualitative studies of all kinds (including mixed methods studies), as well as grey literature. This decision enables us to identify and review all existing published literature evaluating the effectiveness of interventions that support NGN transition into critical care clinical settings. Qualitative studies must assess the effect of the intervention on the transition narratively. We will not place any language or date restrictions on the search. Translation services available at our institution will be used for publications available in languages other than English, French, and Greek when necessary. The latter will enable us to address some of the limitations of prior reviews related to the transition of NGNs and improve the strength of conclusions drawn from the review with a more global perspective.

### Search strategy

An experienced medical information specialist developed and tested the search strategy using an iterative process in consultation with the review team. The strategy was peer reviewed by another senior information specialist prior to execution using the PRESS Checklist. Using the OVID platform, we searched Ovid MEDLINE® ALL, including Epub Ahead of Print and In-Process; Other Non-Indexed Citations, Embase Classic + Embase, and PsycINFO. We also searched CINAHL and Education Source on Ebsco and the Nursing and Allied Health and ERIC databases on Proquest. This protocol also received external review as part of a national funding competition (Canadian Institutes of Health Research Fall 2018 Project Grant awarded (January 23, 2019)). See Table 1 for the search strategy for MEDLINE. Grey literature will be identified using a targeted approach. Specifically, we will search websites of international critical care associations, such as the American Association of Critical Care Nurses, the Canadian Association of Critical Care Nurses, and the European Federation of Critical Care Nurses. Based on our knowledge of this topic and discussion with our clinical partners on this project, these sources are likely to yield the most relevant grey literature.

**Table 1:**
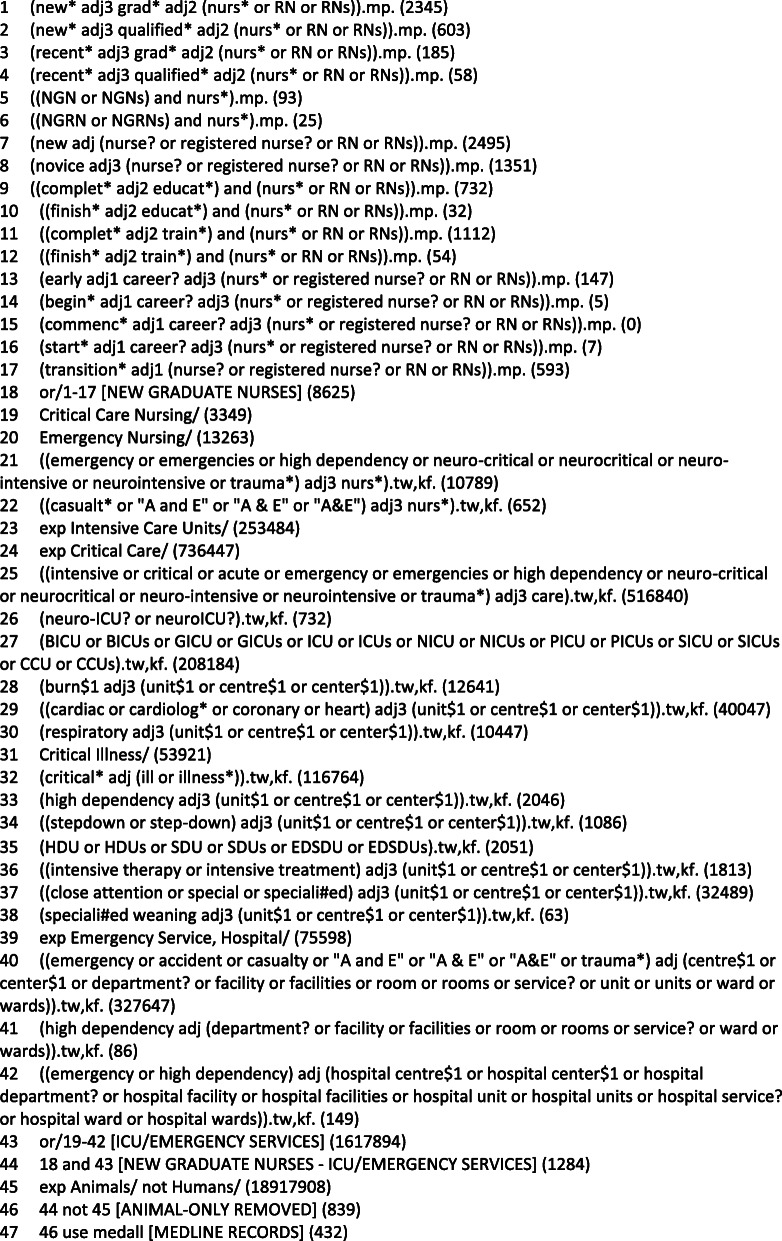
Search strategy—OVID MEDLINE

### Identifying relevant literature

Screening will be undertaken using Covidence®, an online citation screening tool. This software program allows for complete screening of citations, it clearly displays differences in reviewers’ ratings, and helps mitigate issues pertaining to inter-rater reliability. All members of the team are familiar with this software. The selection process will include four steps: (1) removal of duplicates, (2) screen of title and abstract for congruence with eligibility criteria, (3) full-text screen for congruence with eligibility criteria, and (4) hand search of reference lists of included articles. Two team members will independently assess the retrieved citations (steps 2 to 4).

### Methodological quality

Two reviewers will independently assess the quality of each included study using the Joanna Briggs Institute Quality Appraisal Forms (Checklist for Randomized Controlled Trials, Checklist for Quasi-Experimental Studies, Checklist for Cohort Studies, Checklist for Case Control Studies, Checklist for Case Series, Checklist for Analytical Cross Sectional Studies, Checklist for Qualitative Research), and a third reviewer will be consulted as needed if discrepancies cannot be resolved between the first two reviewers. Given the emergent nature of the topic and the descriptive focus of this review, we will not exclude studies based on quality. Instead, we will summarize the appraisal results narratively to report on the state of science in this area.

### Data extraction

Two members of the team trained in review methodologies will independently extract data from all included studies using a standardized extraction form, which we will pilot test to ensure usability with two included studies. The finalized forms will be used to extracting the following data: (1) study identification—authors, publication year, country, province/state, study design; (2) participant characteristics—gender/sex, nursing education, critical care education, sample size, years of nursing experience, years of critical care experience, nursing role; (3) specific operational definition used to identify “new graduate nurses” within the studies; (4) intervention characteristics using the Template for Intervention Description and Replication Checklist (TIDieR) [29]; and (5) outcomes (effect on NGN transition and other author-identified outcomes). For quantitative studies, this will include statistical results about the effectiveness. For qualitative studies, this will include themes/categories (subthemes/subcategories), author interpretations, and supporting quotes that speak to the effect of the intervention on the transition into critical care practice for NGNs narratively.

### Data synthesis

To *identify* interventions, we will aggregate multiple reports on individual interventions so that each intervention (rather than each study) is a unit of interest and create an inventory of discreet interventions. To *describe* interventions, we will use synthesis tables and narrative summaries to report on the intervention characteristics, benefits, and challenges. To assess *effectiveness*, we will first determine whether the interventions and outcomes are amenable to meta-analysis [30]. Effectiveness will only be assessed for studies with adequate information and we anticipate heterogeneous interventions and outcomes. However, should we obtain a subset for meta-analysis, we will assess statistical heterogeneity and pool the results using random-effects methods [30]. We will report on the relative risk (with 95% confidence intervals), absolute risk reduction, and the number needed to treat (i.e., number of people who need to use the intervention) for all types of interventions amenable to meta-analysis. For interventions that cannot be pooled, we will report on outcomes narratively and descriptively using the authored identified primary and secondary outcomes. When the effect is reported qualitatively (i.e., narratively), we will use a simple content analysis approach [31] where we will identify patterns in the reported data across studies. Findings from quantitative and qualitative data will be presented separately to provide a more complete understanding of the topic of interest. Finally, given that the operational definition of “new graduate nurse” is not consistent in both research and professional spheres, we will report on the definitions used to operationalize this phenomenon within the studies. We will tally and report the frequency of use for each operational parameter and, if possible, identify the most commonly used.

### Review quality

We will follow the Preferred Reporting Items for Systematic Reviews and Meta-Analyses (PRIMSA) statement [32], a checklist of 27 essential items for transparent reporting of systematic reviews. We will also use the AMSTAR-2 (https://amstar.ca/docs/AMSTAR-2.pdf) tool, an 11-item checklist (developed as a quality appraisal tool for systematic reviews) to ensure our review meets quality standards and to avert any possible deficiencies in the conduct and reporting of our review [33].

## Discussion

Supporting NGN into critical care areas such as the ICU and ED is important, particularly in light of a broader nursing shortage. This review will identify, describe, and assess the effectiveness of interventions that support NGN transition into these identified areas of specialty nursing practice and will provide an essential foundation for developing strategies to support NGN transition.

### Limitations

It is possible that we will need to complete a descriptive analysis because the interventions eligible for inclusion are likely heterogeneous. Second, it is possible that we will not be able to report complete information due to partial reporting on the identified interventions in primary studies. In order to address these limitations, we have designed our analysis and reporting to include a narrative summary of the available information. We will also contact study authors to request missing or incomplete information. In the instance that we encounter unforeseen circumstances where we need to deviate from our protocol, we will document and report the change in the completed review. Finally, as with all reviews, misinterpretation of the original research or data extraction errors is possible. To minimize this risk, two team members will independently engage in these activities, and discrepancies will be discussed to reach consensus.

End of review knowledge translation activities will include a peer-reviewed publication of the review along with presentations to our knowledge users, the Canadian Association of Critical Care Nurses, and the National Emergency Nurses’ Association.

## Supplementary information

**Additional file 1.** PRISMA-P 2015 Checklist.

## Data Availability

Given that this is a systematic review, there will be no primary data set including primary data; however, the data used in the synthesis will be pulled from published literature that is available.

## Abbreviations

AMSTAR-2: A MeaSurement Tool to Assess systematic Reviews
ED: Emergency departments
IC: Intensive care units
NGN: New graduate nurse
PICO: Population, Intervention, Comparison, Outcomes
PRISMA-P: Preferred Reporting Items for Systematic Reviews and Meta-Analyses Protocols PROSPERO International Prospective Register of Systematic Reviews
